# Candidate Molecular Biomarkers for the Non- Invasive Detection of Colorectal Cancer using Gene Expression Profiling

**DOI:** 10.30699/IJP.2021.132385.2475

**Published:** 2021-03-02

**Authors:** Mohammad Shafiei, Mahdi Alemrajabi, Ali Najafi, Amir Homayoun Keihan, Masoud Reza Sohrabi

**Affiliations:** 1 *Molecular Biology Research Center, Systems Biology and Poisonings Institute, Baqiyatallah University of Medical Sciences, Tehran, Iran*; 2 *Gastrointestinal and Liver Disease Research Center (GILDRC), Firoozgar Hospital, Iran University of Medical Sciences, Tehran, Iran*

**Keywords:** Biomarker, Colorectal cancer, HLTF, SEPT9

## Abstract

**Background & Objective:**

olorectal Cancer (CRC) is the third most common cancer after prostate (breast in women) and lung cancer; it is also the third cause of cancer deaths reported in both men and women in 2020. Currently, the most commonly used diagnostic tools for CRC are colonoscopy, serological methods, and other imaging techniques. Despite the benefits and abilities of these methods, each of them has disadvantages that reduce its functionality and acceptance. The aim of this study was identifying specific and non-invasive genetic biomarkers to diagnose colorectal cancer.

**Methods:**

In this study, changes in the expression of *HLTF* and *SEPT9* genes were evaluated by Real Time PCR in blood and tissue samples of CRC patients. A total of 100 samples (50 Blood and 50 Tissue samples) were evaluated with a definite diagnosis of CRC in Firoozgar Hspital, Tehran, Iran, in 2018. The QPCR method was used to compare the expression of candidate genes between the patients group and control group in both samples. Sensitivity and specificity of the test were examined using ROC curve analysis.

**Results:**

The results showed a significant down-regulation in the expression of both selected genes in tissue and peripheral blood in the various stages of the CRC. The sensitivity and specifity of both genes was about 80%.

**Conclusion:**

The findings showed that the two candidate genes can be suggested as specific biomarkers for diagnosis of CRC using the peripheral blood as a non-invasive method. For a definite conclusion, more research is needed.

## Introduction

Colorectal cancer (CRC) is the third most commonly diagnosed cancer among both men and women in the United States (1). In 2020, almost 147,000 individuals were diagnosed with CRC, and about 53,000 died from the disease globally, while somewhat 17,000 of the cases were younger than 50 (2). Despite many efforts for early detection of CRC, the mortality rate is high. Therefore, a specific and accurate biomarker is needed for patient screening in the onset of cancer of suspected people (3). The most commonly used screening methods are a colonoscopy, CEA marker, and Guaiac-based fecal occult blood test/fecal immunochemical test (gFOBTs/FITs) (4-6) (7, 8). During the last decade, the Colonoscopy method has become the preferred screening tool to detect CRC, because it can detect the polyps and cancer tissues with high accuracy, and also removes cancerous polyps (9). But factors like bowel preparation for colonoscopy, fear of the procedure, its side effects (such as perforation of the intestine), need for sedation, cost and pre- and post-procedure time have reduced the desire of patients for this procedure (10, 11). The other diagnostic tools like CEA, gFOBT, and FIT are underutilized because they have low sensitivity and unsatisfying performance (8, 9). Hence, non-invasive and accurate methods are necessary for early detection and diagnosis of CRC. Circulating DNA and RNA of tumoral cells which are released into body fluids such as blood, serum, stool, and CSF offer new opportunities for non-invasive cancer detection (12). Since the peripheral blood is the common environment, targeting the unique and specific biomarker genes that reflect the behavior of tumoral cells and tissues is an important step in biomarker discovery (13). For this goal, cancer gene expression databases and literature mining were used to select the CRC gene markers. Then, we prepared a list of genes, expression of which changes significantly in cancer cells. Using the network and pathway analysis, Helicase like transcription factor (*HLTF*) and SEPTIN 9 (*SEPT9*) genes were selected, since they are more specific than other genes as CRC biomarkers and less involved in the molecular pathways of other diseases and cancers (3, 14-16). *HLTF* is a DNA helicase protein homologous to the SWI/SNF family that involves maintaining genomic stability and regulation of gene expression (17). The *SEPT9* gene is one of the Septins gene family, an evolutionarily conserved family of GTP-binding protein involved in diverse processes including vesicle trafficking, apoptosis, remodeling of cytoskeleton, infection, neoplasia, and neurodegeneration (18). Some studies have been conducted to discover a non-invasive biomarker for CRC based on the Methylation-specific polymerase chain reaction (MSP) technique for epigenetic changes (3, 14, 19). The difference between Real Time PCR and MSP is that it examines the presence of functional genes involved in protein production while the MSP method focuses on methylation. Therfore, for a more accurate outcome we preferred to use Real Time PCR over MSP, considering ites advantage. The aim of this study was to select non-invasive and specific biomarkers for CRC detection by Real-Time PCR method.

## Materials and Methods

**Patients and Sample Collection**

The statistical society of the research was all the patients who referred to Firoozgar Hospital Tehran, Iran, in 2018. The samples wre collected from the patients that were diagnosed with colorectal cancer (based on clinicopathological findings). A total of 50 patients with CRC (25 tissue samples and 25 peripheral blood samples) and 50 healthy controls (25 healthy blood samples and 25 tissue margins (>10 cm away from the tumor)) were collected. None of the patients had undergone preoperative chemotherapy and/or radiotherapy (Except for 6 samples). The control group did not have any history of gastrointestinal diseases or cancers. The patiens’ group's average age was 58 years old, and for the healthy group (control), it was between 40 and 60 years old. The patients’ group comprised 34 males and 16 females, and there were 32 males and 18 females in the control group (Table 1). The study included patients with all stages of colorectal cancer. All patients signed an informed written consent. The study was approved by the Ethical Committee of the Baqiyatallah University of Medical Sciences (IR.BMSU.REC.1396.110).

**Table 1:** Demographic characteristics and staging of the specimens

The average age of specimens	Chemotherapy and radiotherapy´s specimens	Rectum tissue specimens		Colon tissue specimens		Females	Males	Health tissue specimens	Tumor tissue specimens	Health blood specimens	Tumor blood specimens	Total number of specimens
		Tumor	Health	Tumor	Health							
**40-60**	6	9	9	16	16	34	66	25	25	25	25	**100**
Stage	I			II			III			IV		
Tumor tissue specimens		7				9		4			5	
Tumor blood specimens		7				9		4			5	
All		14				18		8			10	

**Gene Selection**

Many reported genes are effective in cancers. Our goal was to select genes that could identify CRCs specifically and non-invasively. For this purpose, all articles related to CRC in the field of genetic and epigenetic diagnosis without time interval were collected. All genes involved in the CRC were identified, and their reported expression status in the articles was surveyed. After that, changes in the expression of genes, shared both in the blood and tissue, were identified. Then, among the reported genes the ones that demonstrated a lesser association with other diseases and cancers were selected. The interactions of these genes and other genes and pathways involved in cancer were examined using STRING and GeneMania biological networks database, and their interaction network was reconstructed using Cytoscape network analysis software. Then the network was analyzed and clustered. The selected genes were those separated into different clusters. Finally, Primers were selected from the Primer Bank database for two selected genes and control genes. To ensure primers' quality and specificity, the Primer-Blast program and the Oligoanalyzer software were used and the specificity, and secondary structures of primers were examined (Table 2).

**Table 2.** Primer sequences for Real-Time PCR

*SEPT9*	Forward	GCAGAGCGGCTTGGGTAAAT
	Reverse	**ATATCGTGCGTGATGGACTTG**
*HLTF*	Forward	**TGGATGGTGTCACGGGAAAAT**
	Reverse	**TGGTCGGTCCTTCTCAGAAAAA**
GAPDH	Forward	**CTCTCTGCTCCTCCTGTTCG**
	Reverse	**ACGACCAAATCCGTTGACTC**

**Quantitative Real-Time PCR**

Total RNA was extracted from tissue using the trizol-chloroform method and from blood samples using RNA extraction kit (GeneAll Company, South Korea) according to the manufacturer's protocol. The extracted RNA concentrations were measured using a NanoDrop spectrophotometer (Thermo Scientific, US). Then, cDNA was synthesized using GeneAll cDNA synthesis kit (South Korea). 

The Real-time PCR was performed using QIAGEN's Real-time PCR cycler, the Rotor-Gene Q (Germany). The 25 μL PCR included, 13 µL master mix of Amplicon company of Denmark, 2 µL forward and reverse primer (Takapuzist, Iran), 4 µL sterile water and 6 µL cDNA. All reactions were duplicated and the reactions were incubated in a 72-well optical plate at 95°C for 15 min as hot start and 95°C for 20 seconds, 60°C for 20 second and 72°C for 20 seconds for 40 cycles. The Ct data was determinated using default threshold settings.

**Statistical Analysis**

In this study, the ΔΔCt method was used to compare the gene expression between treatment groups and controls. Ct values of the target genes were normalized to the reference gene. Sensitivity and specificity of the test were examined using ROC curve analysis. In all tests, P-value <0.05 was considered to indicate a statistically significant difference. SPSS version 22 (SPSS Inc., Chicago, IL., USA) was used for all statistical analyses. ANOVA and T-test were used to calculate the significant level of the observed difference between the groups. The gene expression difference was calculated using Genex 6 software and drawn using Graph pad prism 7 software.

## Results

**Gene Selection**

**Figure 1:**
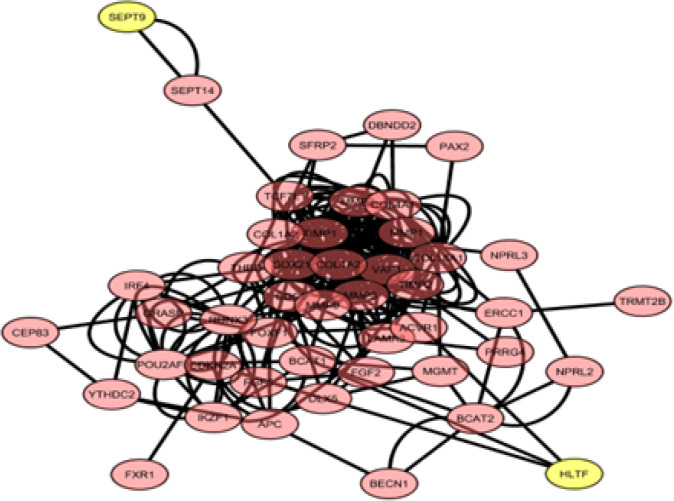
The gene network of CRC master gene list generated using Cytoscape software

*HLTF* and *SEPT9* genes were the best choices because their expression changes were reported in the tissue and peripheral blood. The analysis of the interaction network of selected effective genes in CRC demonstrated that the two genes are clustered as separated cluster and less associated with other diseases and cancers. The interaction network of the effective genes in the CRC is shown in Figure 1.

**The Quantity of Gene Expression**

**Figure 2:**
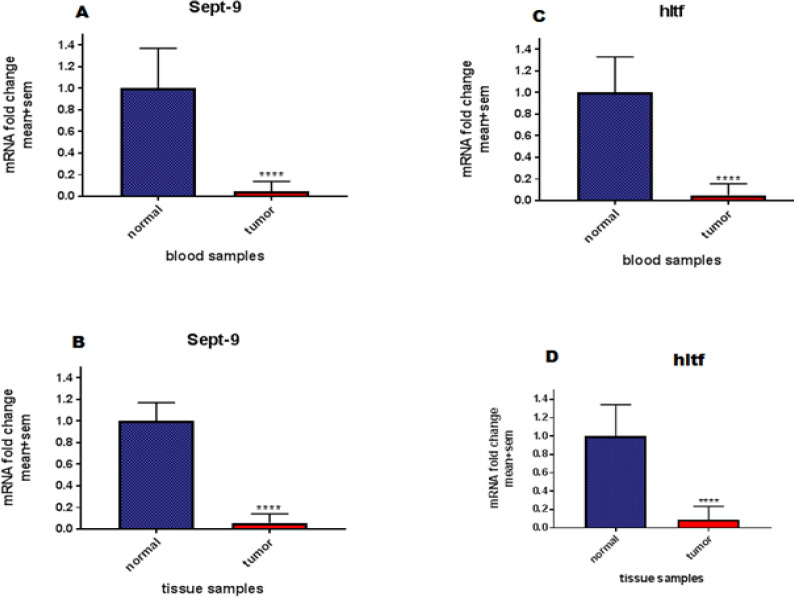
The Expression changes of candidate genes in both cancer and normal groups (blood and tissue samples)

Our study showed a reduction of expression in the cancer group compared with the normal group in the expression of the *SEPT9* gene in blood samples (Fold change: 0.046 and *P*<0.0001). Also, the cancer group compared to the normal group in the *HLTF* gene of blood samples, showed the reduction of expression (Fold change: 0.051 and *P*<0.0001). The expression changes of *SEPT9* gene in tissue samples, revealed the reduction of expression in the cancer group compared to the normal group. (Fold change:0.058 and *P*<0.0001).*HLTF* gene expression changes in tissue samples demonstrated the reduction of expression in the cancer group compared with the normal group (Fold change:0.089 and *P*<0.0001). The results showed the down-regulation of *HLTF* and *SEPT9* genes in both blood and tissue (Figure 2).

As shown in Table 3, we studied the expression changes of *SEPT9* and *HLTF* genes in blood and tissue of both men and women (who underwent or didn’t undergo chemotherapy), as well as in the colon and rectal individually. According to the obtained values in Table 3, the reduction of gene expression in the patients compared to normal samples was obvious.

**Table 3:** Gene expression changes of SEPT9 and HLTF genes

	Source	Sex	Rate (patient/normal)	Rate with chemotherapy	Rate with non-chemotherapy	In tumoral colon	In tumoral rectum
*SEPT9*	Blood samples	Men	Fold change:0.06 and *P*<0.0001	Fold change:0.04 and *P*<0.0001	Fold change:0.06 and *P*<0.0001	Fold change:0.05 and *P*<0.0001	Fold change:0.06 and *P*<0.0001
		Women	Fold change: 0. 07 and *P*<0.0001				
	Tissue samples	Men	Fold change:0.03 and *P*<0.0001	Fold change:0.04 and *P* <0.0001	Fold change:0.06 and *P* <0.0001	Fold change:0.58 and *P* <0.1278	Fold change:0.07 and *P* <0.0001
		Women	Fold change:0.27 and *P*<0.0283				
*HLTF*	Blood samples	Men	Fold change: 0. 03 and *P*<0.0001	Fold change:0.03 and *P*<0.0001	Fold change:0.06 and *P*<0.0001	Fold change:0.05 and *P*<0.0001	Fold change:0.05 and *P*<0.0001
		Women	Fold change: 0. 03 and *P*<0.0001				
	Tissue samples	Men	Fold change:0.13 and *P*<0.0254	Fold change:0.15 and *P*<0.0001	Fold change:0.08 and *P*<0.0001	Fold change:0.07 and *P*<0.0.0001	Fold change:0.12 and *P*<0.0001
Women	Fold change:0.04 and *P*<0.0001

**Figure 3:**
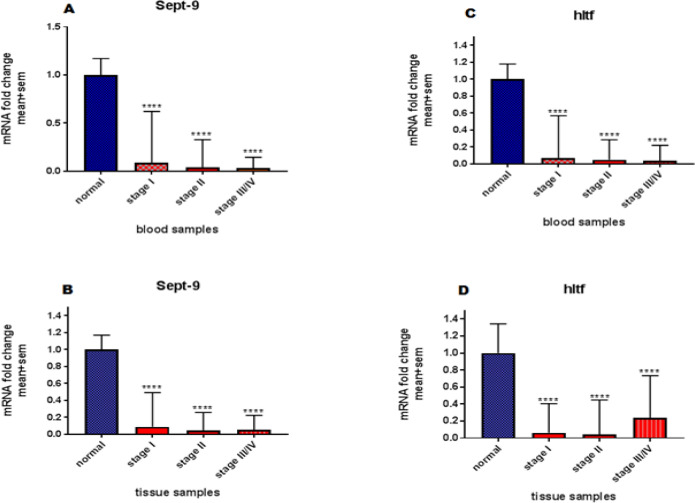
The expression in different stages in blood and tissue cancerous samples

The results obtained from examining the expression changes of both genes in different stages of the CRC demonstrated that the expression changes of *SEPT9* gene in blood samples from low to high stages had a significant decreasing trend. So in the higher stages of CRC the rate of expression reduction was greater than the low stages. The same pattern was observed for tissue samples of this gene, so the expression reduction in higher stages was high, but only in Stage 2 the reduction rate was more than the Stage 3 and 4. The expression changes of the *HLTF* gene in the blood, was like the *SEPT9* gene, so that coordinated by increasing the degree of CRC the reduction of expression in high stages has been most visible. Also in the tissue samples, this pattern was observed so that the reduction of expression in Stage 3 and Stage 4 was more than Stage 1 and 2. The expression of both genes in different stages was illustrated in Figure 3.

The expression changes of these genes in colon and rectum samples was also independently evaluated. The expression changes of *SEPT9* gene in blood samples of patients was evaluated. We observed a reduction of expression in tumoral colon samples compared with normal colon samples (Fold change:0.05 and *P*<0.0001) and also about tumoral rectum samples compared with normal rectum samples, the reduction of expression was observed (Fold change:0.06 and *P*<0.0001). So the reduction of expression in tumoral colon samples was more than the tumoral rectum samples. The expression changes of *HLTF* gene in blood samples of patients demonstrated that the tumoral colon samples had a reduction of expression compared with normal colon samples (Fold change:0.05 and *P*<0.0001); we also saw an approximately similar result in tumoral rectum samples compared with normal rectum samples (Fold change:0.05 and *P*<0.0001). Our findings revealed that the reduction of expression in colon samples was close to that of rectum samples. We also analysed the expression changes of *SEPT9* gene in tissue samples of patients and found that the tumoral colon samples have a reduction of expression compared with normal colon samples (Fold change:0.58 and *P*<0.1278) ) and in the tumoral rectum samples the reduction of expression was observed (Fold change:0.07 and *P*<0.0001). Consequently, the reduction of expression in tumoral rectum samples was more than the tumoral colon samples. About the expression changes of *HLTF* gene in tissue samples of patients, our results demonstrated that the reduction of expression in tumoral colon samples was more than the normal colon samples (Fold change:0.07 and *P*<0.0.0001) ) and also in the tumoral rectum samples the reduction of expression was more than the normal rectum samples (Fold change:0.12 and *P*<0.0001). Thus, the reduction of expression in colon samples was more than the rectum samples.

**Figure 4:**
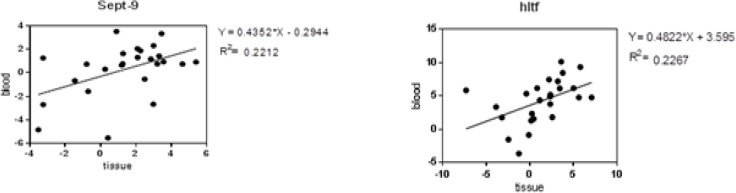
*SEPT9* gene correlation (*P*=0.0177( and *HLTF* gene correlation (*P*=0.0161( between blood and tissue with regression analysis

The results of *SEPT9* and *HLTF* genes correlation between blood and tissue with regression analysis was statistically significant that has been shown in Figure 4.

**Figure 5:**
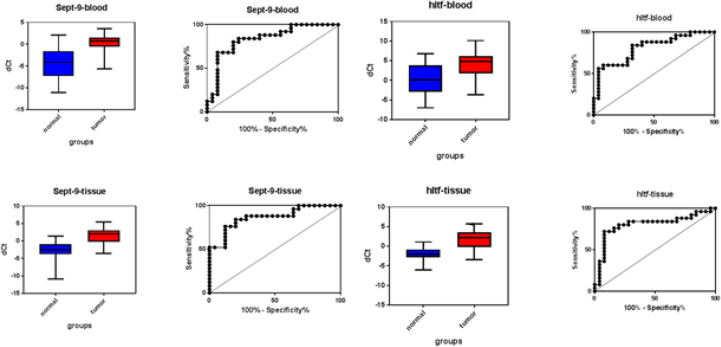
The role of *SEPT9* and *HLTF* genes as biomarkers in the blood and tissue samples

The role of biomarkers for detecting the CRC in tissue and blood specimens was determined by drawing the Roc curve and determining the cutoff point and its sensitivity and specificity. To draw the Roc curve, Δct was used. The results were shown in Figure 5. The sensitivity and specificity of both genes in blood and tissue were studied. These values for the *SEPT9* gene in the blood were 80% sensitivity and 80% specificity (*P*<0.0001) and in tissue were 84% sensitivity and 80% specificity (*P*<0.0001). The *HLTF* gene sensitivity and specificity were respectively 84% and 68% in blood (*P*=0.0002) and were respectively 80% and 80% in tissue (*P*=0.0002).

## Discussion

Screening of colorectal cancer in the early stages of the disease through precise and powerful diagnostic tools reduces the mortality of this complex disease (20). Colonoscopy is currently used as the best diagnostic tool for the CRC, but so many patients do not choose it, because of aggression and other complications. Other diagnostic methods, such as gFOBT and CEA marker, are not sufficiently precise (21). Today researchers are focusing on the potential of genetic and epigenetic biomarkers. Many of these diagnostic targets can be traced and examined in tissue and body fluids such as blood, serum, urine, and stool and used as a non-invasive and accurate diagnostic method (22). Our aim in this study was to investigate the expression changes of *HLTF* and *SEPT9* genes in the tissue and peripheral blood of patients with colorectal cancer and ultimately find a specific and non-invasive diagnostic biomarker for this disease. Several studies have been conducted to investigate the role of these two genes as a colorectal cancer biomarker, most of them have been studied only by tissue or blood, to investigate methylation of these genes, the results indicated that these two genes were hyper-methylated in CRC. Studies have also shown that the decline in the performance of these two genes may also be due to changes in the molding of the mRNA (splicing) process and the formation of defective proteins that do not have the necessary domains to function correctly (23-25). In this study, first, the expression changes of these two genes were studied simultaneously in both the tissue and peripheral blood of patients with CRC, and the correlation between them was evaluated; second, the expression changes of these two genes was investigated in terms of functional genes involved in protein production by Real-Time PCR instead of MSP (methylation). 

Based on the results for both *SEPT9* and *HLTF* genes in blood samples we found that the expression of these two genes in tumor samples was lower than normal and the reduction of expression for *SEPT9* gene was more than the *HLTF* gene. Also, the expression of both genes in tissue samples showed a reduction of expression in tumor samples compared to normal samples and the reduction of expression in *SEPT9* gene was more than that of *HLTF* gene in tissue samples, like blood samples. Accordingly, in both CRC blood and tissue samples, the reduction of expression for *SEPT9* gene was more than the *HLTF* gene. These results are consistent with the results of previous studies (26, 27). Based on former studies, the methylation level of these two genes in tissue and blood samples showed that both *HLTF* and *SEPT9* genes were hypermethylated, resulting from methylation in the promoter of these two genes. Therefore we concluded that the low expression of both genes was probably due to the promoter hypermethylation of these two genes. 

Based on the results, the expression changes of the *SEPT9* gene in blood samples of men and women were examined and we observed that the expression of *SEPT9* gene in male patients was more than normal male subjects and also for female patient the expression was reduced compared with the normal female subjects so the reduction of expression in females was more than males. The expression of *SEPT9* gene in tissue samples of female patients compared with normal females, reduced ;the expression of *SEPT9* gene for male patients compared with normal male subjects showed a reduction. As the results demonstrated, the reduction of expression for male patients was more than the female ones. Song* et al. *(2017) reported the reduction of expression for *SEPT9* gene but no significant difference was seen between men and women; that might be due to the type of method used (MSP instead ofReal time PCR) and also the number of samples (1122 samples in comprison with 100 samples in our study) (28). The expression changes of *HLTF* gene in blood samples of female patients compared with normal ones, showed a decrease. Comparing the change in *HLTF* gene expression between male patients and normal men, we observed that the expression of the aforementioned gene in females reduced more than males. Also, the expression changes of *HLTF* gene in tissue samples of female patients compared with normal women indicated the reduction of expression and about patient men compared with normal men, reduction of expression was reported. Hence, the reduction of expression in female patients was more than the male ones.

Philipp* et al. *(2012) claimed that the methylation level of the *HLTF* gene has been reported in women more than men, which is consistent with the results of this study; although two different tools were used to measure the level of gene expression (29).

The expression changes of *SEPT9* gene in blood of chemotherapy and non-chemotherapy samples compared with normal group showed the reduction of expression; this reduction of expression for non-chemotherapy samples was more than the chemotherapy samples.

About *HLTF* gene in blood samples, the chemotherapy samples compared with normal group indicated the reduction of expression. Also, about non-chemotherapy samples compared with normal group, the reduction of expression was observed; in addition the reduction of expression for chemotherapy group was more than the non-chemotherapy group. Nevertheless, the expression changes of *SEPT9* gene in tissue samples, the patients group (chemotherapy, and non-chemotherapy) compared with the normal group revealed the reduction of expression; this reduction of expression was observed more in chemotherapy samples compared with the non-chemotherapy samples. Comparing the expression changes of *HLTF* gene in tissue samples of patients group and the control group (the normal ones) we found out that the expression of this gene has decreased in the patients group. Additionally, we compared the expression of *HLTF * was lower in the chemotherapy group compared with the non-chemotherapy group. However we didn't find any studies to compare our findings with, and can't say the chemotherapy has a positive or negative effect on the expression of these two genes; therefore we suggest further researches in this matter. 

The expression of *SEPT9* in blood samples of patients with CRC indicated that the reduction of expression for colon samples was more than the rectum samples. In tissue samples, expression reduction for colon specimens was lesser than the rectum. On the other hand, the expression changes of *HLTF* gene in both blood and tissue samples indicated more reduction of expression for colon samples compared with rectum samples. In the study of Philipp *et al.*, (2012) the methylation of the *HLTF* gene in the colon samples was more than the rectum samples, which was similar to the results of the present study (29).

Based on the results the sensivity and specifity of both genes in blood and tissue sampels was about 80%. The study of Toth *et al.*, compared the sensitivity and specificity of SEPT9 gene with both the CEA and gFOBT diagnostic methods currently used as the main diagnostic method, which showed the higher sensitivity and specificity for *SEPT9* gene compared with CEA marker and gFOBT method (30).

According to the results obtained from examining the expression changes of both genes in different stages of the CRC in blood and tissue, the expression changes from low to high stages have had a significant decreasing trend. Therfore, in the higher stages of CRC the rate of expression reduction was greater than the low stages. However, there was a difference in Stage 2 while the reduction of expression was more than Stage 3 and 4 for *SEPT9* in tissue samples. In the study of Song *et al.*, the authors claimed that the expression changes of *SEPT9* gene were observed at all stages, and the expression was lesser than the higher stage of the disease, which confirmed the results of this study (31)..

## Conclusion

The findings showed that the two candidate genes can be suggested as specific biomarkers for diagnosis of CRC using the peripheral blood as a non-invasive method. We suggest more thorough researches with more samples for more accurate outcomes.

## Funding and Sponsership

This research did not receive any specific grant from funding agencies in the public, commercial, or not-for-profit sectors.
